# A Mobile App to Support Parents Making Child Mental Health Decisions: Protocol for a Feasibility Cluster Randomized Controlled Trial

**DOI:** 10.2196/14571

**Published:** 2019-08-14

**Authors:** Shaun Liverpool, Helen Webber, Rob Matthews, Miranda Wolpert, Julian Edbrooke-Childs

**Affiliations:** 1 Faculty of Life Sciences University College London London United Kingdom; 2 Create Health Bristol United Kingdom

**Keywords:** shared decision making, mental health, child, adolescent, parents, technology

## Abstract

**Background:**

Shared decision making (SDM) is recognized as a person-centered approach to improving health care quality and outcomes. Few digital interventions to improve SDM have been tested in child and adolescent mental health (CAMH) settings. One such intervention is Power Up, a mobile phone app for young people (YP), which has shown some evidence of promise that YP who received Power Up reported greater levels of SDM. However, even though parents play a critical role in CAMH care and treatment, they often feel excluded from services.

**Objective:**

This protocol is for a pilot trial to determine the feasibility of a large-scale randomized trial to develop and evaluate a Web app called Power Up for Parents (PUfP) to support parents and promote involvement in CAMH decisions.

**Methods:**

A 2-stage process, consisting of the development stage and pilot-testing stage of the initial PUfP prototype, will be conducted. At the development stage, a qualitative study with parents and clinicians will be conducted. The interviews will aim to capture the experience of making CAMH decisions, preferences for involvement in SDM, and determine situations within which PUfP can be useful. At the pilot-testing stage, up to 90 parents and their clinicians will be invited to participate in the testing of the prototype. Parents will be randomly allocated to receive the intervention or be part of the control group. This study design will allow us to assess the acceptability and usefulness of PUfP in addition to examining the feasibility of a prospective randomized trial. Clinicians’ perceptions of the prototype and how it has influenced parents’ involvement in SDM will also be examined.

**Results:**

Recruitment began in January 2019 and is scheduled to last for 10 months. Interviews and baseline data collection are currently in progress. To date, 11 CAMH sites have been recruited to take part in the study. It is anticipated that data collection will be completed by October 2019.

**Conclusions:**

The lack of parents’ involvement in CAMH care and treatment can lead to higher rates of dropout from care and lower adherence to therapeutic interventions. There are significant benefits to be gained globally if digital SDM interventions are adopted by parents and shown to be successful in CAMH settings.

**Trial Registration:**

ISRCTN Registry ISRCTN39238984; http://www.isrctn.com/ISRCTN39238984

**International Registered Report Identifier (IRRID):**

DERR1-10.2196/14571

## Introduction

### Background

Worldwide, up to 20% of children and adolescents suffer from a disabling mental illness [1,2]. In England alone, 1 in 8 (12.8%) of 5 to 19 year olds have at least one mental health disorder [3]. As a result, families are faced with many decisions, such as how, when, and where to seek help [4]; agreeing on treatment options when more than 1 treatment option is available [5,6]; agreeing on the goals of treatment [7,8]; and agreeing on the diagnostic tests [9]. However, making decisions for young people (YP) with mental health problems can be challenging, as evident by the high levels of disagreement between parents, YP, and clinicians [10-21]. Researchers and practitioners suggest that the implementation of shared decision making (SDM) in child and adolescent mental health services (CAMHS) can be one approach to reduce treatment disagreements [22] and successfully manage the decision-making process that involves balancing multiple perspectives [23,24].

SDM is recognized as a person-centered approach to improving health care quality and outcomes and has been advocated across many health settings, including child and adolescent mental health (CAMH) [23,25,26]. SDM is defined as the communication process that allows service users and service providers to collaborate when making care and treatment decisions [27]. Although there is an increasing demand to include SDM in health care in support of a person-centered approach, attempting to do this in CAMH settings has been met with particular challenges [28].

In adult settings, decisions are *usually* made between the patient and the clinician or, in the case of a triad, the carer is usually another adult. In CAMH settings, the SDM process is unique as it involves a sometimes-complex triad relationship [27,29] between clinicians, children, and parents. Previous studies have mainly focused on the dyad relationships between physicians and patients; therefore, the areas where triad relationships exist have been less understood. When implementing SDM in CAMH, clinicians are forced to moderate highly stressed parents and children and have complex conversations [23].

Researchers highlight common emotional states such as anxiety, distress, sadness, and worry among families involving a child with a mental illness [30-33]. Parents as primary care providers must adopt the responsibilities of caring for a child with mental challenges, which affects their own quality of life [34-36]. Yet, many studies show that parents feel stigmatized and excluded from services [35,37]. Parents feel as if they lack the necessary support from services to help them with supporting their sometimes-unstable child. However, the SDM literature is scarce on whether these emotional factors positively or negatively impact parental decision-making involvement.

As parents are expected to play a critical role in care and treatment, for example, as copatients (family therapy) or as cotherapists (cognitive behavioral therapy), or be the direct focus of the intervention (parent training) [38,39], it is crucial that these parents are involved in the decision-making process. Studies show that involving service users in CAMH care and treatment decisions is associated with improved health outcomes [40] and higher satisfaction with services [41]. Therefore, parents need support to play an active role in the decision-making process.

Improving SDM in CAMH settings can be accomplished when there is an understanding of the factors impacting how parents make or wish to make intervention or care decisions, and when clinicians are able to offer the necessary support for families to be involved in this type of decision making process. SDM includes the notion of a medical encounter as a *meeting of experts*—the physician as an expert in medicine and the patient/parent as an expert in his or her own life, values, and circumstances [42]. British law states that decisions should be made with the child’s best interest at heart [43]. Therefore, the decision needs of parents and children should be addressed to accomplish quality decisions, where the quality of the decision is judged as *good* if there is consistency with the decision maker’s own values and satisfaction with the decisions made while participating in SDM [44].

### Rationale

The prevalence and burden of CAMH on the National Health Service (NHS) is substantial, and supporting large numbers of families at face-to-face sessions can be a challenge [45]; therefore, the use of digital technology can increase access to interventions [46,47]. Mobile technology (ie, mobile phones, tablets, and laptops) use has been on the rise and is estimated to reach 6.1 billion users by 2020 [48]. This offers the opportunity to take advantage of mobile health (mHealth). mHealth is the general term for the use of mobile phones and other wireless technology in clinical practice [49]. Despite the growing number of mHealth apps, the level of awareness and usability of such apps by patients are reported as still relatively low. Nevertheless, the majority of those who use health apps find them to be beneficial and helpful for living a healthier lifestyle [50].

Power Up is an app, co-designed with YP, to empower YP to take an active role in SDM. Power Up has received positive findings, with YP reporting greater levels of SDM after the intervention period [51]. Building on Power Up, this study aims to involve end users in the development of an intervention, called Power Up for Parents (PUfP), to promote SDM and support parents of children and YP accessing CAMHS.

### Research Questions

The primary research aim for this feasibility study is to develop and investigate whether it is feasible and acceptable to conduct a prospective randomized controlled trial (RCT) of an evidence-based mobile app to promote SDM in families accessing CAMHS.

The following research questions will be addressed:

1. Is PUfP acceptable and useful for parents and health care professionals?
2. What is the eligibility, participant consenting rates, adherence, and engagement rates of participants using PUfP?
3. Are the outcome measures appropriate and acceptable for a prospective RCT?
4. What are the potential barriers and enablers to conducting a prospective RCT?
5. Which data collection procedures are appropriate and acceptable?
6. What is the scope of the pilot data collected from users and nonusers of PUfP?
7. Can the feedback from PUfP users be used to further refine the prototype for the prospective RCT?

## Methods

### Design

This a 2-stage study involving a development stage (stage 1) where the intervention will be user-tested by clinicians and parents, and suggestions will be obtained for usage and upgrading of the prototype. The pilot-testing stage (stage 2) is a 3-arm, cluster randomized pilot trial with parents accessing CAMHS.

### Study Setting

The study team has identified CAMH sites from 18 NHS trusts throughout England. The study team identified and agreed to use 9 London and 9 non-London sites. CAMHS is being used as a broad term for all services that work with children and YP who are experiencing mental health challenges. However, the focus centers around, but is not limited to, specialist CAMHS, where children and YP receive services from a multidisciplinary team that includes psychologists and psychiatrists.

### Intervention: Power Up for Parents

The PUfP app is an amended version of the original Power Up app that supports and promotes SDM in CAMH settings. The original Power Up is a mobile app used by YP to empower and encourage them to take an active role in the decision-making process [52,53].

**Figure 1:**
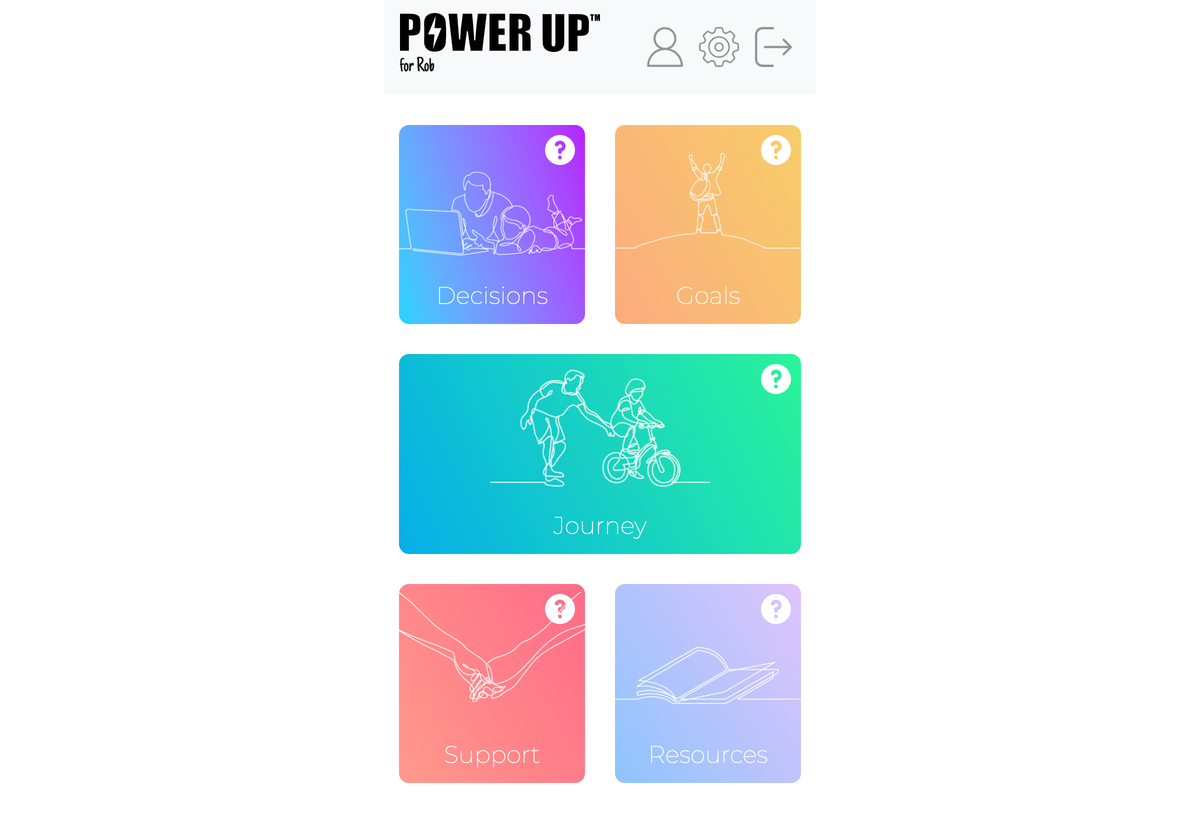
The home screen.

The development of the PUfP prototype was guided by the Workbook for Developing and Evaluating Patient Decision Aids [54] and the Ottawa Decision Support Framework [55]. The prototype (see Figure 1) was informed by a scoping review of existing parent-targeted SDM interventions used in CAMH. A further systematic review was conducted to inform the content of PUfP. We consulted the National Children’s Bureau Families Research Advisory Group (FRAG) to obtain input on the study design and how to improve the intervention before the study began. A steering committee involving parents with experience of having a child with mental health problems and the digital lead at the Anna Freud National Centre for Children and Families (AFNCCF) also offered input on the design of PUfP. The content will be screened by various parent groups and professionals before being used in the pilot study. The overall structure of the app’s content is as follows.

#### Decision

**Figure 2:**
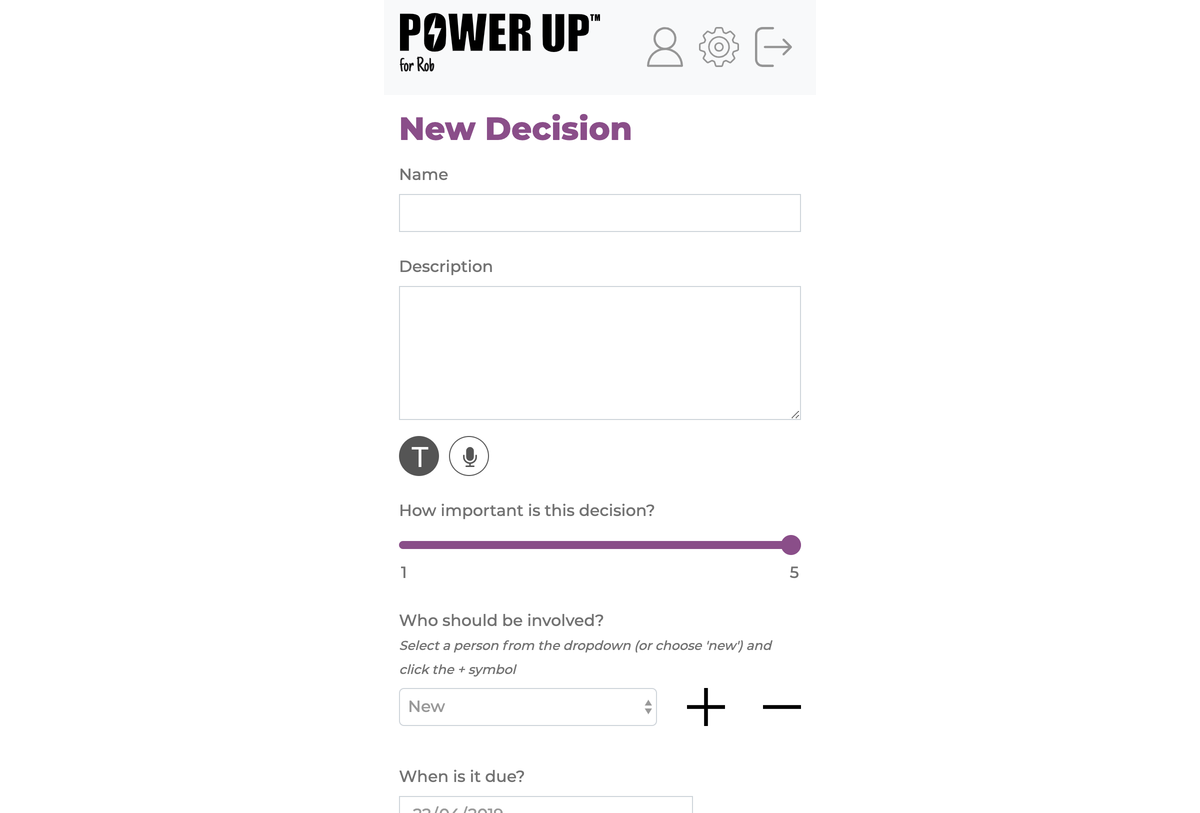
The decision tab.

This is a decision aid that guides users to seek information about treatment options and the benefits and risks of each option, to track decisions, and to record where more information or support is needed. In addition, as this is a triad relationship, users will be prompted to involve others in the decision-making process by seeking preferences from clinicians, child, or other appropriate persons (see Figure 2).

#### Goal

**Figure 3:**
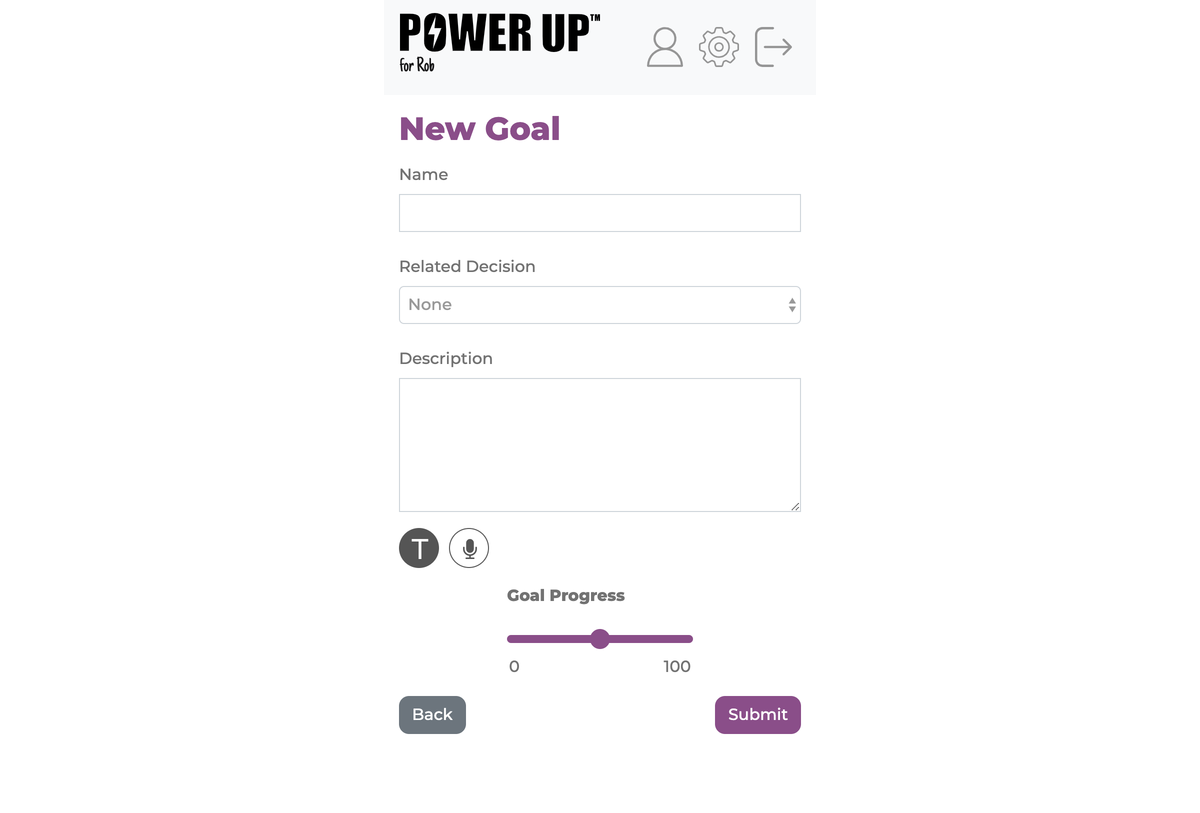
The goal tab.

This feature can be used in sessions or between sessions to record and track goals as they are discussed with health care professionals and the child. This will allow users to plan and record any questions or concerns they have so that they can address them at the next session (see Figure 3).

#### Journey

**Figure 4:**
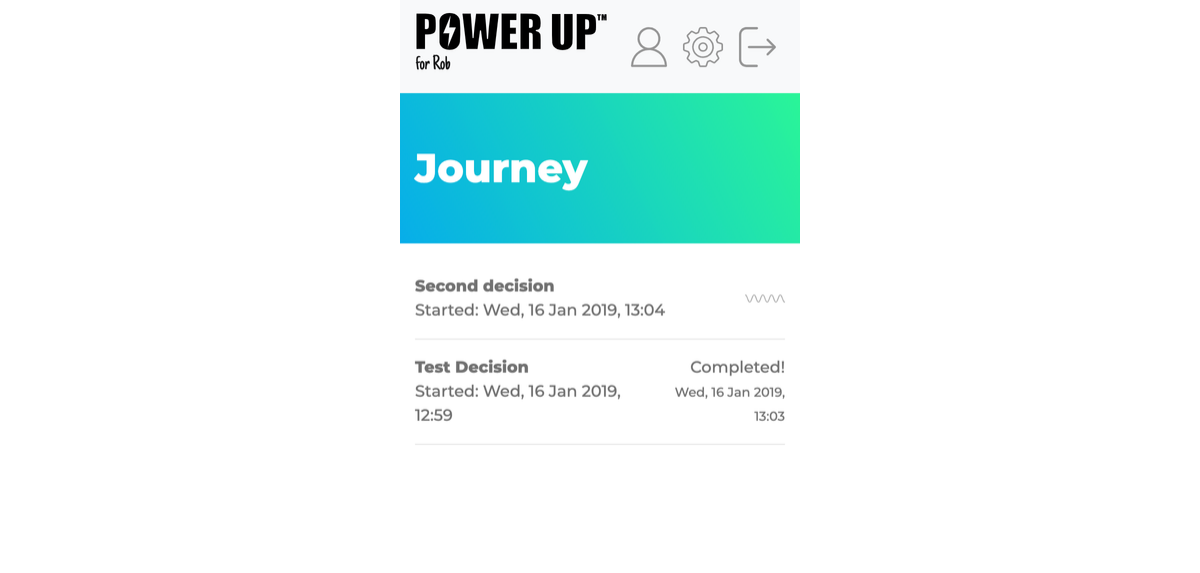
The journey tab.

This feature allows parents to reflect on their emotions or issues that may be affecting the decision-making process. A parent can decide to share this content with the child and the clinician, and it can be used during and within sessions to keep track of the decision-making journey from user readiness to outcomes. Expectations, experiences, and reflections can all be recorded here using the diary function (see Figure 4).

#### Support

**Figure 5:**
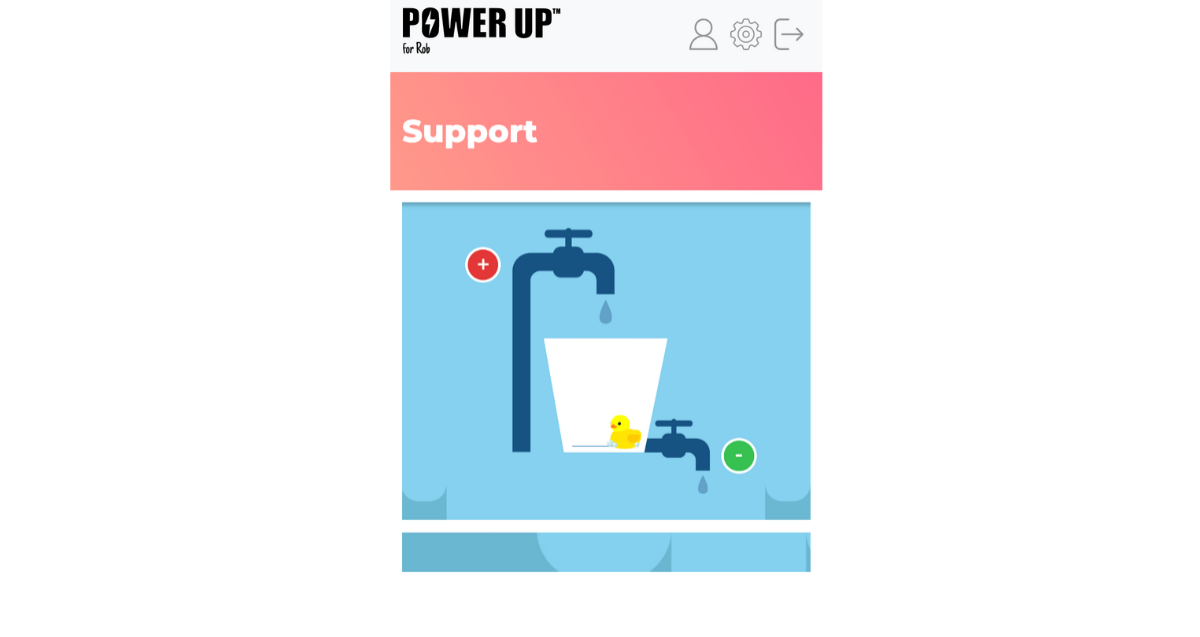
The support tab.

This section will host a tool to allow parents to identify and express views about various stressors affecting the decision-making process. Users will be able to think about things that are stressful and explore ways to manage these. They can track feelings about decisions and explore where additional emotional support is required (see Figure 5).

**Figure 6:**
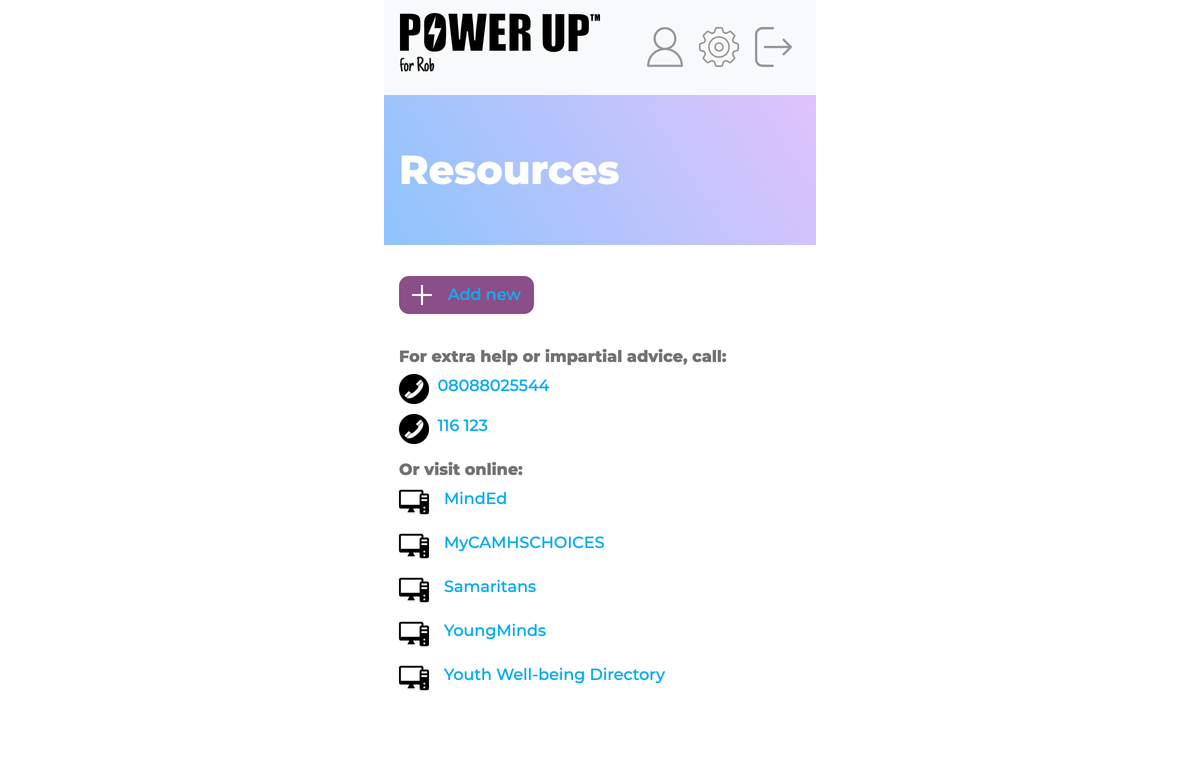
The resources tab.

#### Resources

This section includes useful contact details that can signpost users to further support and guidance. Parents can also upload their own resources to help with the decision-making process and include contacts that they find most helpful (see Figure 6).

### Recruitment

#### Clinicians

All clinicians at the selected NHS sites will be invited to an information session where they will receive information about the study and be given the opportunity to further assist in developing the inclusion criteria for parent participants and inform the recruitment process. This will allow interested clinicians to identify themselves to the research team and become early active participants in the trial. Clinicians for the purpose of this study will support the recruitment process by identifying parent participants within their practice and also, as participants themselves, to inform the development of PUfP and provide feedback on its impact.

#### Parents

On the basis of the inclusion/exclusion criteria agreed at the information session, clinicians will strategically scan their patient list and select suitable participants. At a subsequent meeting with the families, clinicians will solicit interest in participation by sharing some information about the aims of the study. If the family expresses interest in taking part, their contact details will be added to the site’s database of potential participants. The key person at the site will supply the researcher with the database of contact details to invite the parents to participate.

Posters will be placed at participating NHS sites, and parents can also voluntarily contact researchers to express interest in participating. Various parent groups will also advertise the study; therefore, parents may also contact researchers without being identified by clinicians. In addition, the research team will visit the selected CAMHS to recruit participants on clinic days. All parents will be screened to ensure they meet the inclusion criteria. The preliminary inclusion/exclusion criteria are given below.

The inclusion criteria included the following:

1. Over the age of 18 years
2. No known diagnosed mental health issues
3. Ability to speak and understand English
4. Parent of at least one young person (>11 years) attending CAMHS

The exclusion criteria included the following:

1. Concurrent and/or involvement in other research that is likely to interfere with the intervention
2. Parents or guardians in cases where the child/young person is being treated under a section of the Mental Health Act

#### Patient and Public Involvement

Patient and public involvement (PPI) will be conducted throughout the development of PUfP. The opinions and guidance of parent experts and clinicians will be obtained through consultations on the content and the design of the app. We conducted a 3-part consultation session with the FRAG. First, an email consultation round was conducted where the FRAG provided input on the value of PUfP and identified groups of parents that the research team should target for recruitment. Then, the study design and an example of how the intervention might be used were presented to the group. The pros and cons of various modalities for PUfP were discussed along with general thoughts and concerns on the study design. At the final consultation, parents further discussed how service users could use and benefit from the PUfP in practice.

### Procedure and Materials

NHS sites were identified through consultations with supervisors and other researchers at the AFNCCF and University College London (UCL). CAMHS were recruited from across England, and a key contact person was identified at each site. The contact person will circulate information about the study to all clinicians. Then clinicians will be invited to an information session where they will receive further information about the study. The team will then begin to identify suitable participants already accessing their services based on the inclusion/exclusion criteria further developed at this session. This will be a transparent process where clinicians will scan their patient list and select suitable participants.

Clinicians will then inquire if the selected families are interested in the study and add them to the site’s database of interested participants. Each clinician can continue to add names to the database, and the key contact will provide the updated list of interested participants with their respective contact details as new participants are recruited.

In addition, researchers will attend clinics to distribute flyers and recruit onsite volunteer participants. Parent support groups at the CAMHS services will also be approached, and parents will be given the opportunity to volunteer as participants after information about the study is shared. These forms of recruitment will be guided and informed by PPI participants (parent networks) whose expertise is being sourced to inform the recruitment process.

#### Stage 1 (Development Stage)

This stage will involve semistructured interviews and focus groups to obtain qualitative data for the design and content of the PUfP prototype. All participants (ie, clinicians and parents) will be sent information sheets and consent forms in advance of the interviews and focus groups. In addition to gathering parents’ experiences of decision making in CAMHS, an existing prototype of PUfP will be presented, and suggestions for content and prototype upgrades will be obtained. Focus groups are expected to last up to 90 minutes, and interviews (phone or face to face) to last up to 1 hour. The aim is to achieve saturation or involve a minimum sample of 12 interviews or 2 focus group discussions per group [56,57]. After the first 10 interviews, saturation for this study is reached once no new material emerges after further 3 consecutive interviews [58].

In the focus groups and interviews, participants will review the current prototype on materials supplied by the researcher. Feedback on all aspects of the prototype will be requested, and questions about the additional support needs and recommendations for the upgrade will be asked. In addition, the usefulness of the intervention and preference for modality will be sought. At the end of the focus groups and interviews, participants will be debriefed and advised to contact researchers with any further questions or suggestions via our contact details previously given on the information sheets.

#### Stage 2 (Pilot-Testing Stage)

The 18 sites identified will be randomly assigned to either control or 1 of the 2 intervention groups. Intervention group 1 (IG1) will receive the prospective version 1 of PUfP, which includes the *Support* and *Resources* features. Intervention group 2 (IG2) will receive version 2 of PUfP without these 2 features. The cluster randomization was completed independently of the research team, using R software guided by the balance algorithm [59]. Participant-level randomization will not be conducted for this study and parents at all selected sites will have a chance to participate in the study. The Standard Protocol Items: Recommendations for Interventional Trials (SPIRIT) diagram (see Table 1) and flow diagram (see Multimedia Appendix 1) illustrates the pathway through the trial, based on the trial protocol (version 1.3, November 14, 2018) approved by the NHS Research Ethics Committee and the Health Research Authority.

Stage 2 involves reinviting previous participants and obtaining informed consent from all the participants. Participants will be identified and recruited in identical ways as in the development stage. Participants will then be assigned to use the prototype and give feedback on usefulness, usability, and acceptability of the intervention. Up to 90 parents, and their respective clinicians, will be invited to participate in this stage with approximately 30 being allocated version 1 of PUfP, another 30 being allocated version 2, and the remaining 30 as the control group, receiving no app but subject to the same battery of questionnaires and treatment as usual. A sample ranging from 10 to 30 per arm should allow for calculations of feasibility and standardized effect sizes that are small to medium [60,61]. Power calculations, based on repeated measures, within – between comparisons among 3 groups for a single measure across two occasions, indicated that a total sample size of 30 would provide a power of 0.8 to detect an effect size of 0.3. Participants will be assigned to an intervention or control group based on the site from which they have been recruited to avoid contamination.

Participants will meet with the researcher at a time convenient to them, to complete a battery of baseline questionnaires, which consists of SDM measures, the experience of service, and decisional conflict measures. Participants will have the choice to complete these online or using paper and pencil. Depending on which group the participants belong to (IG1 and IG2), they will receive help to access the app and be given a guided tour of the app. The parent will then go away and use the app as much as they need to. Participants will complete follow-up measures about 3 months after or at dropout/discharge (whichever comes first).

**Table 1:** SPIRIT diagram for the Power Up for Parents feasibility trial.

Time point		Study period				
		Enrollment	Pretest	Intervention	Posttest	End of Study
Eligibility screen		X^a^	—^b^	—	—	—
Informed consent		X	—	—	—	—
Allocation		X	—	—	—	—
**Interventions**		—	—	—	—	—
	Intervention Group 1	—	—	X	X	X
	Intervention Group 2	—	—	X	X	X
	Control Group	—	—	—	—	X
**Assessments**		—	—	—	—	—
	Feasibility outcomes	X	X	X	X	X
	CPS-P^c^	—	X	—	X	—
	PSDM-Q-Parent^d^	—	X	—	X	—
	STAI-AD^e^	—	X	—	X	—
	DCS^f^	—	X	—	X	—
	ESQ^g^	—	X	—	X	—
	PSSUQ^h^	—	—	—	X	—

^a^Schedule and time commitment for trial participants.

^b^Not applicable.

^c^CPS-P: Control Preferences Scale for Pediatrics.

^d^PSDM-Q-Pediatric Shared Decision-Making Questionnaire.

^e^STAI-AD: Spielberger State Anxiety Inventory Form for Adults.

^f^DCS: Decisional Conflict Scale.

^g^ESQ: Experience of Service Questionnaire.

^h^PSSUQ: Post-Study Usability Questionnaire.

Clinicians will also complete an adapted version of the Control Preferences Scale (CPS) so researchers can obtain their perspective on changes in the amount of parental involvement in the child’s care and treatment decisions. It may also be important for clinicians to report any changes in the length of appointments or missed appointments and improvements in a child’s mental health.

At the end of the pilot-testing phase, participants will share their opinions on the study and, more specifically, on the intervention used and will then be debriefed and thanked for their participation.

### Outcome Measures

#### Stage 1 (Development Stage)

##### Demographic Characteristics

Participants will be asked both categorical (eg, gender, ethnicity, first language, and relationship to child) and continuous (eg, age) demographics. Demographic data on the patient population will also be collected. This will determine the profile of the families and health care professionals participating in the study.

##### Interview Topic Guide

The focus groups and interviews will follow a topic guide that aims to allow participants to review the current prototype of PUfP and provide feedback on all aspects of the prototype. Feedback will inform content and design while exploring the decision-making approaches of parents and clinicians. The topic guide will also explore parents’ emotions and how they impact the decision-making process. These interviews and focus groups are expected to provide preliminary qualitative input on the acceptability and usefulness of PUfP.

#### Stage 2 (Pilot-Testing Stage)

##### Participation Numbers

The number of sites that are approached and the number of sites agreeing to take part will be recorded, in addition to the number of participants agreeing to take part in the study. The proportion of participants completing various parts of the study (ie, consent, pretest, intervention, and posttest) will also be recorded. The app usage rates will also be collected using Google Analytics software.

##### The Control Preferences Scale for Pediatrics

The Control Preferences Scale for Pediatrics (CPS-P) [62] is an adaptation of the CPS [63]. This tool was originally developed to measure “the degree of control an individual wants to assume when decisions are being made about medical treatment” [64]. The CPS-P consists of 5 different scenarios describing different levels of control preference in decision making. The original scale has been tested in a variety of populations, ranging from the general public to highly stressed groups. The CPS has proven to be a clinically relevant, easily administered, valid, and reliable measure of preferred roles in health care decision making [64]. Permission was obtained to modify and reproduce the CPS-P. Therefore, we also adapted the questionnaire to obtain clinicians’ perspectives on how parents preferred to be involved in decisions. Clinicians will be asked to select 1 of 5 statements on whether “the parent leaves all mental health care and treatment decisions about the child to the practitioner” or “the parent shared responsibility for the mental health care and treatment decisions about the child with the practitioner.”

##### Pediatric Shared Decision-Making Questionnaire

The 9-item Pediatric Shared Decision-Making Questionnaire (PSDM-Q-9)—Parent (modified) version measures the extent to which parents are involved in the process of decision making from the perspective of the parent. The measure was developed for use in research and clinical practice. This tool is commonly used for the purposes of evaluation and quality improvement in health care. This measure is being applied in the case of preference-sensitive decisions—that is, when there are several options to be considered before a particular decision is made. This measure has shown face validity and high acceptance. Internal consistency yielded a Cronbach alpha of .938 in a test sample [65].

##### Spielberger State Anxiety Inventory Form for Adults

The Spielberger State Anxiety Inventory Form for Adults (STAI-AD) is a 40-item self-reported questionnaire commonly used as a measure of trait and state anxiety. It is used in research as an indicator of caregiver distress. The STAI-AD internal consistency coefficients ranged from .86 to .95; test-retest reliability coefficients ranged from .65 to .75 over a 2-month interval [66]. In addition, test-retest coefficients for this measure in another study are rated as highly significant with an intraclass correlation coefficient ranging from .39 to .89 [67].

##### Decisional Conflict Scale

The 16-item Decisional Conflict Scale was developed to elicit information concerning the decision maker’s (1) uncertainty in making a choice; (2) modifiable factors contributing to the uncertainty, such as lack of information, unclear values, and inadequate social support; and (3) perceived effective decision making [68]. This scale quantifies factors that contribute to uncertainty both during the process and at the outcome. Previous studies have shown that the psychometric properties of the scale are acceptable, and it is feasible and easy to administer [68].

##### Experience of Service Questionnaire

The Experience of Service Questionnaire (ESQ) measures service satisfaction and is widely used in CAMHS in the United Kingdom. The ESQ consists of 12 items and 3 free-text sections looking at what the respondents liked about the service, what they felt needed improving, and any other comments. The satisfaction with care construct will be obtained by adding up items 1 to 7, 11, and 12 [69]. These constructs are important to the study as the SDM process and outcome may impact parents’ perception of service satisfaction. On the basis of literature reviews of SDM [70], the research team agreed that the following questions also assess the key components of SDM: (1) I feel that the people who have seen my child listened to me; (2) It was easy to talk to the people who have seen my child; (4) My views and worries were taken seriously and (6) I have been given enough explanation about the help available here [40].

##### The Post-Study System Usability Questionnaire

The Post-Study Usability Questionnaire (PSSUQ) is a 19-item usability quantification survey developed in 1992 by the IBM Design Centre. The PSSUQ is generally used to quantify the usability of websites, apps, or any software or hardware that users interact with. The questionnaire is a series of statements describing the app that users agree or disagree with using a Likert scale [71]. For this study, the more general term *system* was replaced with the word *app*, and therefore, questions were more targeted, such as “I was able to complete the tasks and scenarios quickly using this app.” This measure aims to further assess the usability, appropriateness, acceptability, and feasibility of the overall intervention.

#### Planned Analysis

##### Stage 1: Development Stage

Data collected from focus groups and interviews will be analyzed using thematic analysis [72]. Themes will inform the development stage of the intervention by providing the relevant material to inform the content and design of the final version of PUfP. All interviews and focus group sessions will be audio-recorded digitally and transcribed. The computer package Atlas.ti or NVivo will be used to manage the data. Data will be coded using a combination of a priori themes and emergent themes [73].

##### Stage 2: Pilot-Testing Stage

The data collected will be analyzed with the main aim to reflect on potential improvements to the content of the intervention and informing the overall study design for a prospective RCT. The correlation between demographic data, app use, and outcome measures will be explored to identify target groups. Qualitative and quantitative evaluations will be carried out to examine the acceptability of the intervention as a useful decision aid for parents of children accessing CAMHS. In addition, between-group mean differences at the 2 time points (ie, baseline and follow-up) will be conducted. The standard deviations and intraclass correlation coefficients of the SDM measure will identify the parameters to enable planning for the subsequent trial. The outcome of these analyses will be used to calculate the sample size for the future RCT. Analyses will be conducted using the SPSS software and mostly presented descriptively. The main focus will be on descriptive data, with some exploratory significance testing. The amount of missing data will also be reported for each group.

Outcomes from stages 1 and 2 will be tested against a predetermined set of criteria. Multimedia Appendix 2 summarizes the criteria for assessing the feasibility of a prospective RCT upon completion of the feasibility trial.

#### Risk Register

##### Ethical Approvals, Research Governance, and Study Sponsorship

This research project has been ethically reviewed by the London Surrey Research Ethics Committee and approved by the Health Research Authority (IRAS 236277). This study will also be guided by UCL Ethical Standards, the Declaration of Helsinki (2008), the International Conference on Harmonisation Good Clinical Practice, and conducted in accordance with the Department of Health Research Governance Framework for Health and Social Care (April 2005) and the Data Protection Act (2018).

##### Confidentiality

Interview and focus group recordings will be held securely on a password-protected server at the evidence-based practice unit (using the Data Safe Haven system) until the recording has been transcribed, at which point the recording will be deleted. Transcripts will be held securely under a uniquely identifiable number on the Data Safe Haven system and will be anonymized at the point of transcription. Consent forms will also be kept in secure locked storage, separate from the research data. Anything containing names and contact details will be stored separately and securely from the research data. The data collected during the pilot-testing stage in questionnaire format will only be identifiable by a unique number and will also be kept securely. All paper documents will be kept in secure locked storage, and once the data have been entered into an electronic state, the paper versions will then be shredded and disposed of according to UCL’s standards for disposing of confidential waste. Appropriate access controls will be in place to ensure that access to confidential research information is restricted to the main researcher (SL) and immediate supervisors (JEC and MW). All data will be collected, handled, and stored in accordance with local and national information governance procedures, including the Data Protection Act (2018).

##### Safeguarding

To protect both the participants and researchers, safeguarding procedures of UCL/AFNCCF, in addition to the NHS site procedures, will be strictly adhered to. Safeguarding protocols will be followed if at any point of the research a participant reveals information that suggests he/she is of serious risk to themselves or others. If any participant becomes distressed or too emotional during the interviews, they will be treated with compassion and empathy by trained/experienced researchers and signposted to further help if necessary. The content of the questionnaires have been reviewed to ensure that the standardized measures that are least likely to cause further burden are selected.

##### Recruitment

Parents and clinicians will need to set aside time for participating in interviews/focus groups. The researchers will remain flexible, and parent participants will be offered prospective travel reimbursement where necessary. Participation will remain voluntary to avoid any burden to participants, and all participants at every stage will be required to give informed consent. Details of their role in the study will be given both orally and via the information sheets, and all questions will be addressed. Participants can opt out at any stage of the study if they feel uncomfortable.

##### Intervention

The actual use of the app may become an inconvenience for the parent or clinician. Owing to the co-design approach taken, the features should be something that parents are interested in using. By taking this approach, it is less likely that the use of the app will be seen as an inconvenience. In addition, clinicians are allowed to be flexible with the use of the prototype during sessions and therefore can manage any time strains.

## Results

Recruitment began in January 2019 and is scheduled to last for 10 months. Interviews and baseline data collection are currently in progress, and to date, 11 CAMH sites have been recruited to take part in the study. It is anticipated that data collection will be completed by October 2019.

## Discussion

To our knowledge, this is the first feasibility study to pilot-test an interactive parent-targeted digital SDM tool across CAMHS in England. This 2-stage research project and its findings will inform the development and testing of a parent-targeted SDM Web app to be used in CAMHS. It will target parents/primary caregivers of children seeking various mental health services. PUfP is expected to offer the necessary support parents need when making decisions for/with children with mental health challenges.

One advantage to this study’s approach is that parents and clinicians themselves get to shape a tool that caregivers, clinicians, and other service providers and users may access in the future. Participants may find taking part in research to be rewarding, as they contribute to the development of knowledge that may benefit themselves and others. Parents will feel supported and empowered to make informed choices and feel included in the SDM process alongside their child and the health professionals. This approach coincides with the National Institute for Health and Care Excellence guidelines on service users having the right to be involved in discussions about treatment and care [25]. This research will have implications for the use and implementation of digital interventions within the NHS and future research in the area of SDM as a triad process in CAMHS.

Finally, the findings from this feasibility study will inform the planning of a prospective RCT. The larger study will add to this initial understanding of how the implementation of PUfP can aid in the promotion of SDM in CAMHS while reducing parents' feeling of exclusion from the decision-making process. The prospective RCT may also be able to highlight other areas PUfP can impact, for example, on decreasing waiting times if parents have quicker access to support. This study and the prospective RCT can have research, policy, and practice implications for how SDM is managed in CAMHS with the use of technological interventions.

## Appendix

Multimedia Appendix 1 Go No-Go Criteria.

Multimedia Appendix 2 Flow diagram of the Power Up for Parents (Stage 2) feasibility trial.

## Abbreviations

AFNCCF: Anna Freud National Centre for Children and Families
CAMH: child and adolescent mental health
CAMHS: child and adolescent mental health services
CPS: Control Preferences Scale
CPS-P: Control Preferences Scale for Pediatrics
ESQ: Experience of Service Questionnaire
FRAG: Family Research Advisory Group
IG: intervention group
mHealth: mobile health
NHS: National Health Service
PPI: patient and public involvement
PSSUQ: The Post-Study System Usability Questionnaire
PUfP: Power Up for Parents
RCT: randomized controlled trial
SDM: shared decision making
STAI-AD: State-Trait Anxiety Inventory for Adults
UCL: University College London
YP: young people
