# Supplementary material for: A Mobile App to Support Parents Making Child Mental Health Decisions: Protocol for a Feasibility Cluster Randomized Controlled Trial
Source: JMIR Res Protoc. 2019 Aug 14;8(8):e14571. doi: 10.2196/14571 (PMC6712959; doi:10.2196/14571)
Supplement: Multimedia Appendix 1 [file resprot_v8i8e14571_app1.pdf]

Criteria description	Type	Assessment of readiness	Score
Parents reporting PUfP[a] as acceptable in interviews and outcome measures	People	0%-20% of parents report that PUfP is acceptable	0
		21%-49% of parents report PUfP is acceptable	1
		50%-100% of parents report PUfP is acceptable	2
Parents reporting PUfP as useful in interviews and outcome measures	People	0%-20% of parents report that PUfP is useful	0
		21%-49% of parents report PUfP is useful	1
		50%-100% of parents report PUfP is useful	2
HCPs[b] reporting PUfP as acceptable in interviews	People	0%-20% of HCPs report that PUfP is acceptable	0
		21%-49% of HCPs report PUfP is acceptable	1
		50%-100% of HCPs report PUfP is acceptable	2
HCPs reporting PUfP as useful in interviews	People	0%-20% of HCPs report that PUfP is useful	0
		21%-59% of HCPs report PUfP is useful	1
		50%-100% of HCPs report PUfP is useful	2
Ability to recruit and retain sites	People	0%-19% of sites recruited and retained	0
		20%-49% of sites recruited and retained	1
		50%-100% of sites recruited and retained	2
Ability to recruit and complete baseline measures	People/process	0%-19% of sites recruited and retained	0
		20%-49% of sites recruited and retained	1
		50%-100% of sites recruited and retained	2

Rates of access and usage of PUfP	People/process	0%-19% of parents access and use PUfP	0
		20%-49% of parents access and use PUfP	1
		50%-100% of parents access and use PUfP	2
Ability to retain participants and complete follow-up measures	People/data/process	0%-19% of sites retained and completed follow-up measures	0
		20%-49% of sites retained and completed follow-up measures	1
		50%-100% of sites retained and completed follow-up measures	2
**Aggregate meaning**			**Total score**
Ready to proceed to full RCT[c]	The criteria have been attained		12-16
Ready with some action to be taken	The criteria have not been attained but an existing plan can bring attainment of the criteria or allow the RCT to proceed in the absence of attainment		6-11
Not ready	The criteria have not been attained and there is no existing plan than can bring attainment of the criteria		0-5

[a]PUfP: Power Up for Parents.

[b]HCP: health care provider.

[c]RCT: randomized controlled trial.
