# Supplementary figures and images for: A Mobile App to Support Parents Making Child Mental Health Decisions: Protocol for a Feasibility Cluster Randomized Controlled Trial
Source: JMIR Res Protoc. 2019 Aug 14;8(8):e14571. doi: 10.2196/14571 (PMC6712959; doi:10.2196/14571)

Randomization

Enrollment

Allocation

Follow-up

Analysis

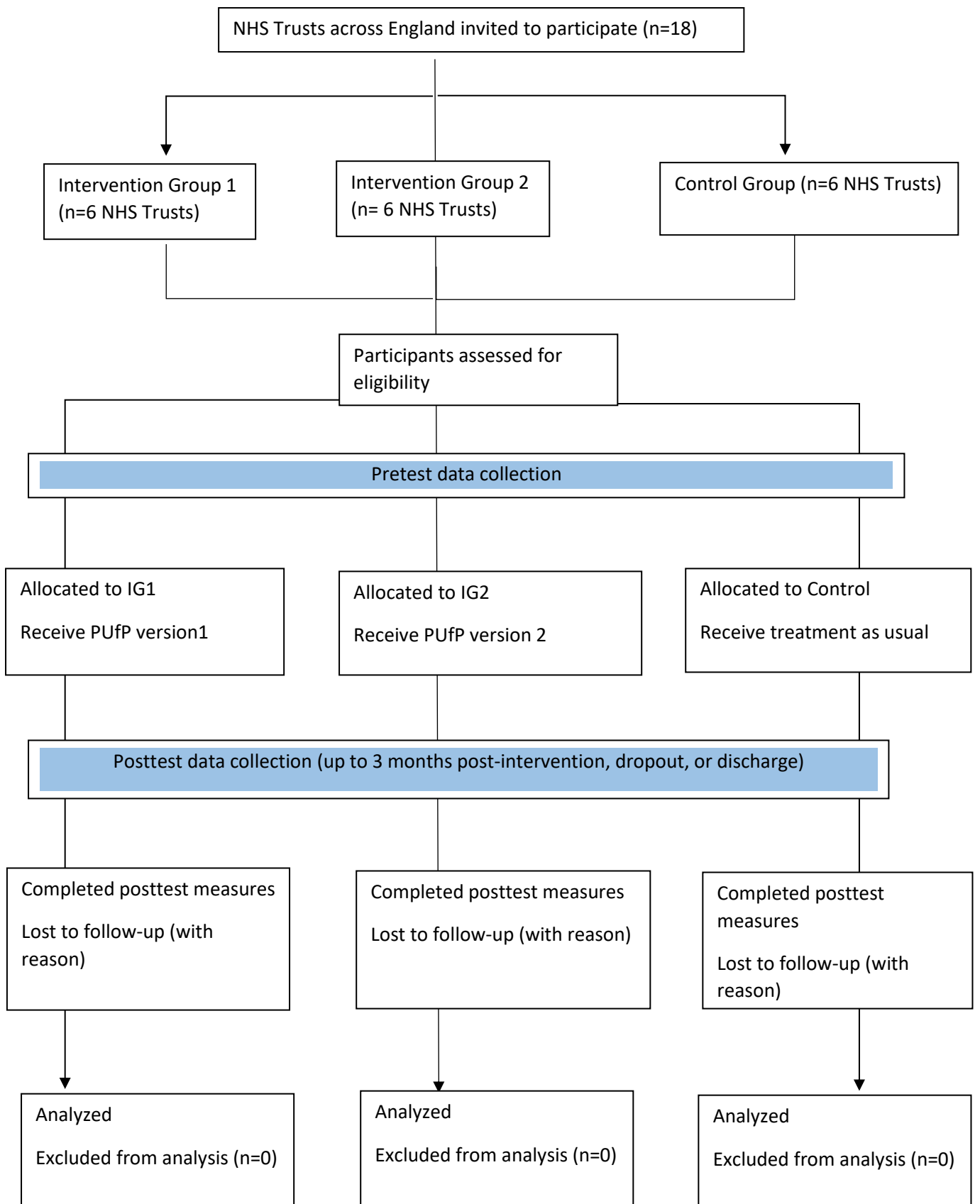

Supplement: Multimedia Appendix 2 [file resprot_v8i8e14571_app2.pdf]
